# Accumulation of Mixed Heavy Metals in Maternal Hair and Risk of Pre-Eclampsia: A Prospective Nested Case–Control Study

**DOI:** 10.3390/toxics13070575

**Published:** 2025-07-08

**Authors:** Thi Ha Luu, Gege Ma, Ming Jin, Xiaojing Liu, Mengyuan Ren, Suhong Gao, Jiamei Wang, Rongwei Ye, Xiaohong Liu, Nan Li

**Affiliations:** 1 Department of Epidemiology and Biostatistics, School of Public Health, Peking University, Beijing 100191, China; 2 Key Laboratory of Reproductive Health, National Health Commission of the People’s Republic of China, Institute of Reproductive and Child Health, Peking University, Beijing 100191, China; 3 Department of Maternal and Child Health, School of Public Health, Peking University, Beijing 100191, China; 4 Department of Obstetrics and Gynecology, Haidian Maternal and Child Care Hospital, Beijing 100101, China

**Keywords:** pre-eclampsia, heavy metals, combined effect, pregnant women

## Abstract

Heavy metals (lead [Pb], cadmium [Cd], arsenic [As], mercury [Hg], manganese [Mn], copper [Cu], zinc [Zn], and iron [Fe]) might be risk factors for pre-eclampsia (PE), whereas their joint effect remains unclear. To address this issue, we conducted a nested case–control study consisting of 49 PE cases and 329 controls from a Chinese prospective birth cohort and divided the participants into low/high and quartile groups based on hair metal concentrations. We used logistic regression models and a weighted quantile sum (WQS) model to investigate the independent and mixed associations between these eight heavy metals in maternal hair and the risk of PE. After multivariable adjustment, high hair Pb was associated with a 2.53-fold increased risk of PE, and significantly higher risks of PE were also observed in quartiles 2 to 4 of Pb and quartiles 3 to 4 of Fe. The WQS model revealed a statistically significant association between maternal co-exposure to all eight heavy metals and the risk of PE, with Pb, As, and Fe presenting the biggest risk. Therefore, high maternal exposure to heavy metals may increase the risk of PE. It is crucial to consider co-exposure to multiple heavy metals throughout pregnancy in future research endeavors.

## 1. Introduction

Pre-eclampsia (PE) is a multi-system pregnancy disorder characterized by new-onset hypertension and end-organ dysfunction that complicates about 3–5% of all pregnancies and remains one of the leading causes of maternal and perinatal morbidity and mortality [1]. The adverse intrauterine environment in women with PE is thought to contribute to increased risk of childhood and adult chronic diseases in offspring [2]. Prenatal exposure to environmental chemicals has been implicated in adverse pregnancy outcomes [3]. In particular, the role of prenatal heavy metal exposure in PE development has attracted much attention in growing epidemiologic studies [4,5,6,7,8].

Previous studies mainly focused on the effects of exposure to single heavy metals such as lead (Pb), cadmium (Cd), mercury (Hg), and arsenic (As), confirming positive associations with elevated risk of PE [4,5,9,10,11,12,13]. Other heavy metals such as iron (Fe), copper (Cu), zinc (Zn), and manganese (Mn) are also involved in blood pressure changes or hypertension during pregnancy [6,14,15,16], indicating potential correlations with PE. The complex interaction of these heavy metals results in discrepant health effects between individual and co-exposure to multiple components [17,18]. However, existing evidence on the mixed effects of multiple heavy metals is greatly limited and inconsistent [6,7,8], so it needs to be given priority in future research for better risk screening for PE.

Since some pregnant populations are usually far away from exposure sources and pollutants containing heavy metals, their potential health effects seem to result from chronic accumulation of heavy metals in vivo. We found the above-mentioned related studies with blood [10,11,13] and urine [9,12] samples to reflect the short-term exposure levels of heavy metals. However, there is no evidence reporting the effects of long-term exposure to heavy metals, which can be well evaluated using hair specimens [19,20]. Therefore, it is important to determine hair heavy metal contents in pregnant women and then assess their individual and mixed effects on PE risk.

Heavy metal pollution is still a major environmental concern in China [21], where heavy metal (i.e., Cd, Zn, and Cu) levels in urban road dust have been found to be higher than the Chinese maximum allowable concentration of potentially toxic elements [22]. Given limited evidence for the role of antenatal heavy metal exposure and their joint exposure in PE, we conducted a nested case–control analysis based on a prospective birth cohort in Beijing, China. This study aimed to identify whether the accumulation of heavy metals in maternal hair affected PE development and whether there was mixed effect of antenatal co-exposure to multiple heavy metals.

## 2. Materials and Methods

### 2.1. Study Design and Participants

This nested case–control study was based on a prospective birth cohort implemented in the maternal and child healthcare hospital in Beijing, China. The original cohort study aimed to investigate the effects of antenatal environmental exposure on pregnancy outcomes. Pregnant women were randomly invited to participate in the cohort if they met the following inclusion criteria: (1) aged 18 years or older; (2) within 20 gestational weeks; (3) being a non-immigrant resident of the county; and (4) decided to deliver in the study hospital. A total of 2731 pregnant women who planned to deliver in this hospital were recruited between October 2017 and October 2018. We excluded 502 women without hair samples or volume depletion, 604 women with missing outcome information, and 42 women with a history of chronic diseases such as hypertension. Among the remaining 1583 participants, 49 women who were diagnosed with PE were classified into the case group. A total of 329 pregnant women were selected as the control group to increase statistical power according to maternal age, pre-pregnancy BMI, parity, ethnicity, education, occupation, and high-quality data collection and experimental records. This study was approved by the Biomedical Institutional Review Board of Peking University (approval number: IRB00001052-17028) on 28 April 2022 and promoted by the China Cohort Consortium with the registration identifier CCC2018112301 “http://chinacohort.bjmu.edu.cn/ (accessed on 20 April 2024)”. All participants completed the written informed consent form.

### 2.2. Data and Sample Collection

On recruitment at the first antenatal examination, a structured questionnaire was used by trained interviewers to collect information on participants’ sociodemographic characteristics. Blood pressure was measured by experienced healthcare professionals, and pre-eclampsia diagnosis was obtained from medical records. Maternal hair was cut as near to the scalp as possible at the time of first antenatal examination. The hair samples were then stored at −20 °C and analyzed in less than one year. A length of 24 cm hair was collected to cover the exposure level during a 2-year period (average growth rate of 1 cm per month).

### 2.3. Measurement of Heavy Metals and Analysis

The method of measuring heavy metal elements in hair has been described in detail in a previous study [20]. Each raw hair sample was cut into ~5 mm fragments and crushed with a grinding machine. An approximately 60 mg sample was mixed with 0.6 mL of concentrated nitric acid and 0.5 mL of ultrapure water, then the mixture was digested in a microwave system for one hour at 100 °C (MARS 6, CEM Co., Matthews, NC, USA). The concentrations of eight heavy metals (As, Cd, Cu, Fe, Hg, Mn, Pb, and Zn) were measured using inductively coupled mass spectrometry (NexION350x; Perkin Elmer, Waltham, MA, USA). The limit of detection (LOD) for each element was as follows: Pb, 0.002 µg/g; Hg, 0.007 µg/g; As, 0.011 µg/g; Cd, 0.0001 µg/g; Zn, 0.005 µg/g; Fe, 0.075 µg/g; Cu, 0.004 µg/g; and Mn, 0.002 µg/g. Elemental concentrations below their LODs were replaced with the respective LOD divided by the square root of two.

### 2.4. Statistical Analysis

The baseline characteristics in the case and control group were compared via Student’s *t*-test for continuous variables and the χ^2^ test for categorical variables. Element concentrations in hair were distributed non-normally and presented as median with interquartile range (IQR). The differences between heavy metal levels in PE cases and controls were analyzed using the Mann–Whitney U test. Pregnant women were classified into two subgroups (low and high) based on the heavy metal concentration at the 50th percentage, as well as four subgroups (quartiles 1, 2, 3, and 4). Logistic regression analyses were performed to estimate the odds ratios (ORs) and 95% confidence intervals (CIs) for PE associated with different exposure levels of heavy metals in both crude and adjusted models. The potential cofounders included maternal age, pre-pregnancy BMI, parity, ethnicity, education, and occupation. The effect of heavy metal co-exposure on PE risk was further assessed based on the WQS regression model with or without adjusting for potential confounders. All statistical analyses were executed using R Studio 4.2.1 and SPSS 23.0. A two-sided *p*-value < 0.05 was considered to be statistically significant.

## 3. Results

### 3.1. Participant Characteristics

Table 1 shows the population characteristics of the pregnant women included in this nested case–control study. Overall, the baseline characteristics (i.e., age, pre-pregnancy BMI, ethnicity, parity, education level, and occupation) were comparable between the PE cases and the controls (All *p* > 0.05).

### 3.2. Levels of Heavy Metals in Maternal Hair

The distributions of eight heavy metals (As, Cd, Cu, Fe, Hg, Mn, Pd, and Zn) in hair based on PE status are shown in Table 2. The detection rates of most heavy metals were almost 100%, except for As (29.6%). The medians (IQRs) of hair Pb and Fe concentrations in PE women were 0.409 (0.284–0.603) and 22.7 (19.8–28.2), significantly higher than those in the controls, which were 0.327 (0.221–0.522) (*p* = 0.016) and 21.0 (16.4–27.0) (*p* = 0.028), respectively. The median concentration of Hg in women with PE was lower than that in the control group, but the difference was not statistically significant (*p* = 0.766). PE women had higher median levels of other heavy metals compared with the controls. However, all differences were not significant (*p* > 0.05).

### 3.3. Associations of Individual Heavy Metals in Maternal Hair and Pre-Eclampsia Risk

The pregnant women were divided into low and high subgroups according to the median heavy metal concentrations. PE women were more likely to have high levels of As, Cd, Cu, Fe, Mn, Pb, and Zn in hair, while both low and high levels of Hg showed similar proportions between PE women and controls. Compared with the low groups, pregnant women who had high hair Pb levels were significantly associated with a more than 2-fold risk of PE after either adjusting for the confounders (OR 2.53, 95% CI 1.31–4.86) or not doing so (OR 2.54, 95% CI 1.34–4.85). A high hair Fe level could also significantly increase the risk of PE, with ORs (95% CI) being 2.06 (1.10–3.86) in the crude model and 2.17 (1.14–4.12) in the adjusted model. There was no statistical significance between high levels of the other heavy metals and an elevated risk of PE (see Table 3).

**Figure 1:**
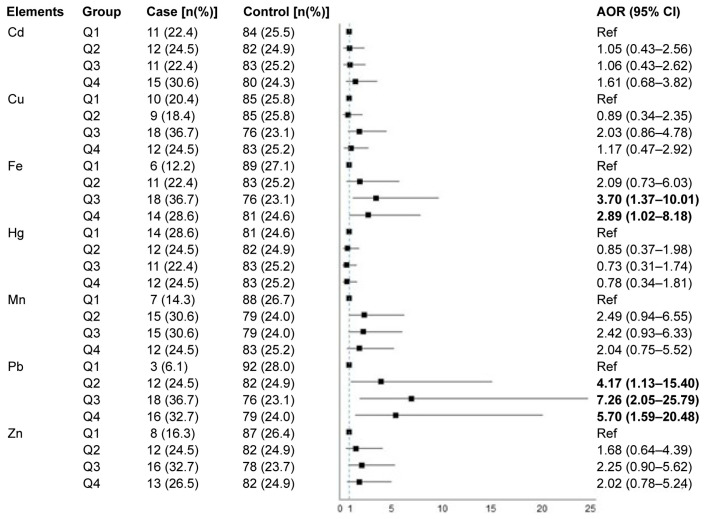
The associations of heavy metals in maternal hair based on quartiles and pre-eclampsia risk. The dashed line is a reference line with OR = 1. The square boxes and solid lines represent point estimates and 95% CIs of the OR, respectively. Bold texts indicate statistical significance. The adjusted model was adjusted for age, pre-pregnancy BMI, parity, ethnicity, education, and occupation. The lowest quartile of heavy metal concentration was used as a reference. Abbreviations: Cd, cadmium; Cu, copper; Fe, iron; Hg, mercury; Mn, manganese; Pb, lead; Zn, zinc; AOR, adjusted odds ratio; 95% CI, 95% confidence interval; Ref, reference.

Element concentration in hair was further classified into quartiles to analyze its dose–response effect on the risk of PE. The pregnant women in the lowest quartile were used as a reference. After being adjusted for the potential confounders, Pb concentrations at high second, third, and fourth quartiles significantly increased the risk of PE, with adjusted ORs (95% CI) being 4.17 (1.13–15.4), 7.26 (2.05–25.8), and 5.70 (1.59–20.5), respectively. Moreover, pregnant women with high hair Fe levels at the third (adjusted OR 3.70, 95% CI 1.37–10.0) and fourth (adjusted OR 2.89, 95% CI 1.02–8.18) quartiles were significantly associated with a greater risk of PE, while the association for hair Fe at the second quartile was not significant. The linear trends were also observed for Pb (*p* = 0.028) and Fe (*p* < 0.050). The other heavy metals were not significantly associated with PE risk (Figure 1).

### 3.4. Mixed Effects of Heavy Metal Co-Exposure in Maternal Hair on Pre-Eclampsia Risk

**Figure 2:**
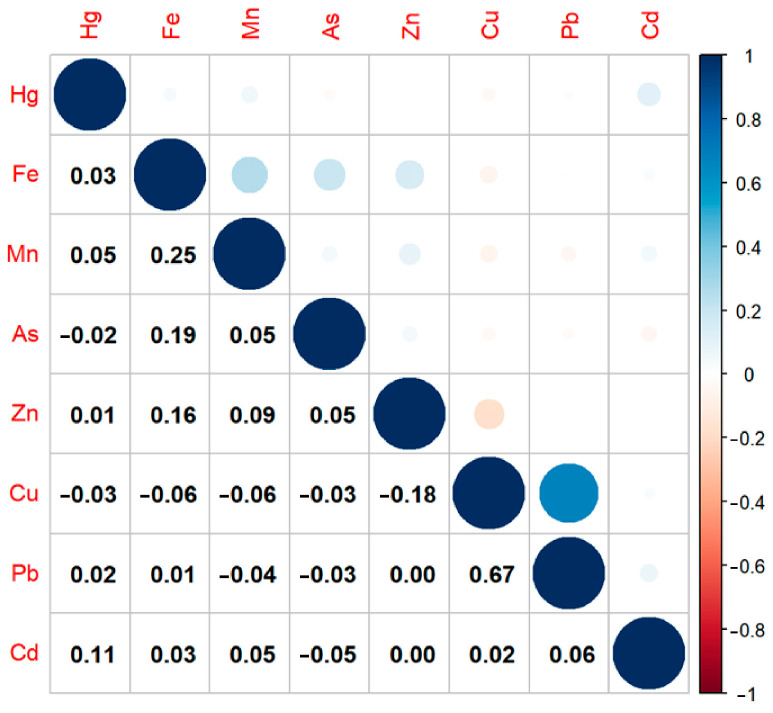
The correlation matrix plots of eight heavy metals. The circle size represents the magnitude of the absolute value of the correlation coefficient. Abbreviations: As, arsenic; Cd, cadmium; Cu, copper; Fe, iron; Hg, mercury; Mn, manganese; Pb, lead; Zn, zinc.

**Figure 3:**
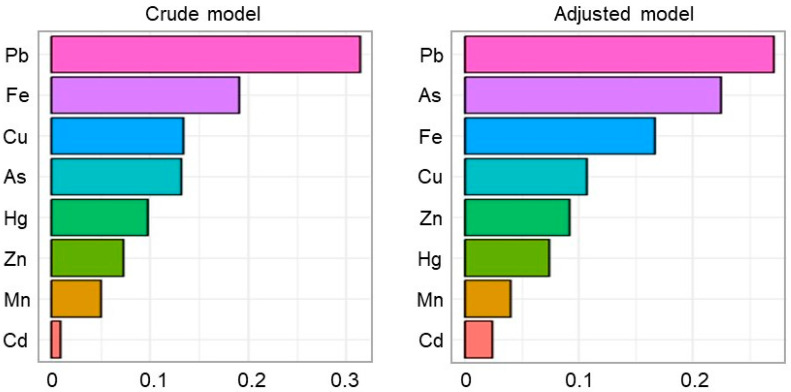
The estimated risk and weighted value of heavy metals for pre-eclampsia via WQS regression analysis. The adjusted model was adjusted for age, pre-pregnancy BMI, parity, ethnicity, education, and occupation. Abbreviations: WQS, weighted quantile sum; As, arsenic; Cd, cadmium; Cu, copper; Fe, iron; Hg, mercury; Mn, manganese; Pb, lead; Zn, zinc.

The correlation matrix plot revealed a significant correlation between Cu and Pb (Figure 2). Therefore, WQS regression analysis was used to address the issue of collinearity between the elements. According to the results of the WQS regression analysis, there was a significant relationship between the WQS index and the risk of PE in both the crude (*p* = 0.032) and adjusted (*p* = 0.015) models. After adjusting for the potential confounders, the index was mostly predominated by Pb (27.1%), As (22.5%), and Fe (16.7%), followed by Cu, Zn, Hg, Mn, and Cd (Figure 3).

## 4. Discussion

We found that PE women had higher concentrations of Pb and Fe than those in the controls, which independently increased the risk of PE. Similar associations were observed in As, Cd, Cu, Mn, and Zn, although these associations were not significant. We further found that co-exposure to the eight heavy metals was associated with the risk of PE, mostly attributed to Pb, As, and Fe.

We compared the concentrations of the eight heavy metals in hair samples of pregnant women in previous human studies (see Table 4). Among PE cases and control women in South Africa, the concentrations of As, Cd, Cu, Fe, Mn, Pb, and Zn were obviously higher than those found in our study [7]. Heavy metals including Cd [23], Cu [23], Fe [23], Hg [24,25,26], Mn [23], Pb [23,25,27], and Zn [23] were also higher in the general pregnant population of other countries. Compared with the above results, Zhao et al. detected Cd, Mn, and Pb concentrations in pregnant women from Guangxi, China, and they found results more similar to ours, though with a slight increase to some extent [28]. In another study conducted in northern China, Cd concentration in pregnant women’s hair was similar to our results. The concentrations of Hg and Pb were slightly lower and higher than those in our study, respectively [29]. Those findings suggested that the Chinese pregnant population has been living away from metal pollution sources, which can be considered to represent normal exposure levels for the general population. In addition, we found that the concentrations of heavy metals changed as the time increased in the same population [30,31,32,33]. The participants in our study were enrolled in the birth cohort from 2017 to 2018, whose accumulation of heavy metals in hair was in accordance with the current situation. We consider that our results can provide more valuable evidence on the effects of cumulative exposure to heavy metals on PE occurrence.

We found a significant association between high levels of hair Pb and an elevated risk of PE. Pb is a well-established risk factor for pre-eclampsia [5]. Previous studies showed that pregnant women with PE have significantly higher concentration of blood Pb than controls [13,34,35,36]; Dawson et al. found that Pb concentrations in amniotic fluid also significantly increased in PE women [34]. Previous studies suggested a positive association between Pb concentration and the risk of PE [6,8,36,37]. Our findings were consistent with previous results [6,8,36,37] and provided additional evidence on the effects of Pb accumulation in hair specimens. Higher Pb levels might induce oxidative stress [38], which could trigger the release of anti-angiogenic factors associated with endothelial dysfunction and plays an important role in the pathogenesis of PE [4]. There have been limited studies about the associations for the remaining heavy metals. We found non-significant associations between As, Cd, Cu, Hg, and Zn in hair and the risk of PE. Similarly, one study conducted in a Chinese cohort did not find associations in As and Cd, albeit with a positive association in Hg [8]. Another cohort study conducted in Egypt between 2016 and 2017 also showed that urinary Hg was associated with a greater risk of PE [9]. A study conducted in Guangdong, China, also found an adverse effect of serum Hg on PE [37]. Previous studies showed that As exposure in various samples did not affect the prevalence of PE [6,7,8,12], which generally supports our findings. In addition, existing evidence on the association with Cd was not consistent across studies using different samples of blood [8,10], hair [7], amniotic fluid [34], and placenta [39]. Some studies revealed significantly elevated levels of Fe [40], Cu [37,41,42,43], Zn [41,42,43], and Mn [36] in blood samples of pregnant women with PE, with a strong association with PE risk. However, contrary results were found in some other studies for serum Cu, Fe, Mn, and Zn [6,44], as well as hair Zn and Cu [7]. The sample sizes in the above studies with negative results were relatively small, which might lead to insufficient statistical power. The inconsistent associations observed in Cd, Cu, Hg, Mn, and Zn needed cautious interpretation, and they should be verified in larger-sized samples with different biological samples and populations.

Multiple heavy metals shared exposure sources, e.g., Fe, Cu, Zn, As, Hg, and Pb exposure from food consumption [45,46,47,48], as well as Mn, As, Cd, Hg, and Pb exposure from cigarette smoking, drinking water, and metal products [49,50,51]. Emerging studies [52,53,54] have proved a joint association of heavy metal co-exposure with varied adverse health outcomes. Although neither our study nor previous studies observed significantly individual effects of some heavy metals on PE development, the joint effect of heavy metal mixture should not be neglected. In this study, we found that eight heavy metals’ co-exposure significantly increased the risk of PE, with contributions to the WQS index by Pb, As, Fe, Cu, Zn, Hg, Mn, and Cd in a multivariate-adjusted model. The results were similar to the findings of a recent case–control study [8] conducted between 2012 and 2016 in Taiyuan, China. Wang et al. detected the concentrations of blood metals in pregnant women; they confirmed that the WQS index, predominated by Cr, Hg, Pb, and As, was positively associated with PE [8]. However, Borghese et al. did not observe overall joint effects for blood metals (Pb, As, Hg, Cd, and Mn) according to a quantile g-computation model in a Canadian cohort between 2008 and 2011 [6]. To our knowledge, our study contributed the first evidence on the joint effects of heavy metal accumulation in hair on the risk of PE. The inconsistency in the results may be explained by the differences in genetic predisposition and biological specimens. More studies are needed to provide a reference for the PE risk assessment of short-term and long-term exposure to heavy metal mixtures.

Our study has some limitations. First, the concentrations of hair heavy metals in our study were slightly lower compared with other biological samples; hair As was especially low due to over two-thirds of the study population being missing. However, there was limited evidence to examine the association of heavy metal and PE risk in populations with low exposure levels. Second, we examined total elements in hair rather than their possible attributions in inorganic, organic, or methyl form. Wells et al. found that methyl and inorganic Hg were associated with higher and lower blood pressures among pregnant women, respectively [55]; it is possible that the conflicting associations affected the integrated effect of total Hg on PE risk that we observed. Finally, the relatively limited number of PE women studied did not allow us to estimate associations of heavy metals for women with different subtypes of PE, which should be given priority in future studies.

This study had several strengths. The nested case–control study was derived from a prospective birth cohort, which could provide strong evidence for the causality between heavy metal exposure and PE risk and allow us to use a small sample size for statistical inference. The participants in our study were exposed to relatively low levels of heavy metals, so the present results played a vital role in PE risk assessment for a general pregnant population. In addition, hair samples were usually used to assess cumulative concentrations of chemical elements. We focused on hair heavy metals to represent their long-term exposure levels to explore actual associations with PE. Since human beings were exposed to multiple metals at the same time rather than a single one, we used a WQS regression model to estimate the joint effect of a mixture of heavy metals. The joint effect that we observed provided reference values to improve the prevention of PE.

## 5. Conclusions

Our results provided supportive evidence that maternal hair Pb and Fe were independent risk factors for PE, and even relatively low accumulation of Pb significantly increased the risk of PE. We are the first to propose that cumulative co-exposure to the eight heavy metals (As, Cd, Cu, Fe, Hg, Mn, Pb, and Zn) in maternal hair plays a significantly deleterious role in PE development. Our study highlighted the importance of estimating individual and joint associations between heavy metals and PE, as well as reinforcing awareness of the need to incorporate multiple metal exposure tests during pregnancy.

## Figures and Tables

**Table 1:** Baseline characteristics of pregnant women based on pre-eclampsia status in China.

Characteristics	Control Group (*N* = 329)	Case Group (*N* = 49)	*p*
Age (years, mean [SD])	31.2 [3.78]	31.8 [3.99]	0.255
Pre-pregnancy BMI (kg/m^2^, mean [SD])	22.3 [3.40]	22.2 [3.33]	0.863
Parity			0.091
Primiparous	262 (79.6%)	44 (89.8%)	
Multiparous	67 (20.4%)	5 (10.2%)	
Ethnicity			0.979
Han	313 (95.1%)	46 (93.9%)	
Minority	16 (4.9%)	3 (6.1%)	
Education			0.741
Postgraduate	87 (26.4%)	13 (26.5%)	
Undergraduate	145 (44.1%)	24 (49.0%)	
Junior college or lower	97 (29.5%)	12 (24.5%)	
Occupation			0.675
Worker/business/services	76 (23.1%)	10 (20.4%)	
Professional and technical staff	107 (32.5%)	13 (26.5%)	
Public official	74 (22.5%)	12 (24.5%)	
Others	72 (21.9%)	14 (28.6%)	

Abbreviations: SD, standard deviation; BMI, body mass index.

**Table 2:** Distributions of heavy metals in maternal hair among pre-eclampsia women and controls in China.

Elements	LOD	DR	Case (*N* = 49)	Control (*N* = 329)	*p*
(μg/g)		*N* (%)	Median (IQR)	Median (IQR)	
As	0.011	112 (29.6)	0.613 (0.231–0.757)	0.530 (0.310–0.915)	0.323
Cd	<0.001	378 (100.0)	0.017 (0.012–0.026)	0.015 (0.010–0.022)	0.157
Cu	0.004	378 (100.0)	11.0 (7.83–14.1)	9.94 (6.99–13.8)	0.329
Fe	0.076	378 (100.0)	22.7 (19.8–28.2)	21.0 (16.4–27.0)	0.028
Hg	0.007	378 (100.0)	0.433 (0.268–0.682)	0.455 (0.290–0.680)	0.766
Mn	0.002	378 (100.0)	0.379 (0.296–0.550)	0.345 (0.226–0.534)	0.078
Pb	0.002	378 (100.0)	0.409 (0.284–0.603)	0.327 (0.221–0.522)	0.016
Zn	0.006	378 (100.0)	256 (204–380)	238 (183–370)	0.176

Abbreviations: As, arsenic; Cd, cadmium; Cu, copper; Fe, iron; Hg, mercury; Mn, manganese; Pb, lead; Zn, zinc; LOD, limit of detection; DR, detection rate; IQR, interquartile ranges.

**Table 3:** Associations between heavy metals in maternal hair and pre-eclampsia risk.

Elements		Case Group	Control Group	Crude OR	Adjusted OR ^a^
		*n* (%)	*n* (%)	(95% CI)	(95% CI)
As					
	Low	31 (63.3)	235 (71.4)	1	1
	High	18 (36.7)	94 (28.6)	1.45 (0.77–2.72)	1.42 (0.75–2.69)
Cd					
	Low	23 (46.9)	166 (50.5)	1	1
	High	26 (53.1)	163 (49.5)	1.15 (0.63–2.10)	1.28 (0.69–2.38)
Cu					
	Low	19 (38.8)	170 (51.7)	1	1
	High	30 (61.2)	159 (48.3)	1.69 (0.91–3.12)	1.57 (0.83–2.98)
Fe					
	Low	17 (34.7)	172 (52.3)	1	1
	High	32 (65.3)	157 (47.7)	2.06 (1.10–3.86)	2.17 (1.14–4.12)
Hg					
	Low	26 (53.1)	163 (49.5)	1	1
	High	23 (46.9)	166 (50.5)	0.87 (0.48–1.58)	0.81 (0.44–1.51)
Mn					
	Low	22 (44.9)	167 (50.8)	1	1
	High	27 (55.1)	162 (49.2)	1.27 (0.69–2.31)	1.33 (0.72–2.46)
Pb					
	Low	15 (30.6)	174 (52.9)	1	1
	High	34 (69.4)	155 (47.1)	2.54 (1.34–4.85)	2.53 (1.31–4.86)
Zn					
	Low	20 (40.8)	169 (51.4)	1	1
	High	29 (59.2)	160 (48.6)	1.53 (0.83–2.82)	1.57 (0.83–2.97)

^a^ Adjusted for age, pre-pregnancy BMI, parity, ethnicity, education, and occupation. Abbreviations: As, arsenic; Cd, cadmium; Cu, copper; Fe, iron; Hg, mercury; Mn, manganese; Pb, lead; Zn, zinc; OR, odds ratio; 95% CI, 95% confidence interval.

**Table 4:** Comparisons of heavy metal concentrations in pregnant women in different regions.

Reference	Region	Study Time	Subjects	No. of	Sample Time	Age	Concentrations of Heavy Metals in Hair (µg/g) ^a^							
				Participants	(gwk) ^a^	(Years) ^a^	As	Cd	Cu	Fe	Hg	Mn	Pb	Zn
The present study	Beijing, China	2024	Control	329	1st trimester	31.2 ± 3.78	0.53 (0.31–0.92)	0.02 (0.01–0.02)	9.94 (6.99–13.8)	21.0 (16.4–27.0)	0.46 (0.29–0.68)	0.35 (0.23–0.53)	0.33 (0.22–0.52)	238 (183–370)
			PE	49		31.8 ± 3.99	0.61 (0.23–0.76)	0.02 (0.01–0.03)	11.0 (7.83–14.1)	22.7 (19.8–28.2)	0.43 (0.27–0.68)	0.38 (0.30–0.55)	0.41 (0.28–0.60)	256 (204–380)
Maduray et al. [7]	South Africa	2017 ^c^	Control	23	/ ^f^	24 ± 5	5.47 ± 2.79	3.75 ± 0.64	78.8 ± 28.2	449 ± 78.4	/	13.6 ± 1.13	58.8 ± 37.0	331 ± 29.7
			PE	43		25 ± 5	7.63 ± 1.32	3.96 ± 0.87	58.9 ± 17.3	614 ± 107	/	13.1 ± 0.95	72.3 ± 19.8	396 ± 48.6
Manduca et al. [23]	Palestine	2014–2015	Total	502	1st trimester	26.9 ± 5.92	0.07 (0.01–1.04)	0.04 (0.00–0.54)	12.70 (1.90–22,700)	14.6 (1.53–868)	0.19 (0.01–2480)	0.72 (0.04–14.2)	1.50 (0.07–331)	284 (34.9–2160)
Trdin et al. [25]	Croatia	2007–2009	Total	222	3rd trimester	30.1 ± 4.8	/	/	/	/	0.51	/	/	/
Palir et al. [26]	Italy	2006–2009	Total	873	2nd and 3rd trimesters	32.7	/	/	/	/	0.77 (0.73–0.81)	/	/	/
Muniroh et al. [24]	Indonesia	2018	Total	118	2nd trimester	29.5 (19–39)	/	/	/	/	0.43 (0.15–8.11)	/	/	/
Black et al. [27]	Britain	1980s	Total	71	37–42	/	/	/	/	/	/	/	0.55 (0–12.1)	/
Zhao et al. [28]	Guangxi, China	2012	Healthy group	57	<20	26.7 ± 4.73	/	0–0.04, 71.4% ^b^	/	/	/	1–1.90, 91.2% ^b^	0–0.68, 73.8% ^b^	/
Kippler et al. [30]	Sweden	1996	Total	655	/	29 ± 4.0	/	/	/	/	0.38 (0.17–1.5) 0.25 (0.03–1.1)	/	/	/
		2019												
Kocyłowski et al. [31]	Poland	2016	Total	108	17.7 ± 5.3	31.4 ± 4.9	/	/	17.3 ± 9.9	/	/	/	/	179 ± 50.1
Sikorski et al. [33]	Poland	1986	Total	104	40 (37–42)	26 (15–42)	/	/	6.55	16.4	/	/	2.14	152
Ripley et al. [32]	Canada	2006–2011	Pregnant women	913	Prenatal	25.5	/	/	/	/	0.84 ^d^ (0.77–0.91)	/	/	/
Zhu et al. [29]	Northern China	2018 ^c^	No ^e^	172	/	/	/	0.02 (0.01–0.03)	/	/	0.13 (0.10–0.17)	/	0.63 (0.35–1.35)	/
			Yes ^e^	84		/	/	0.01 (0.01–0.02)	/	/	0.15 (0.11–0.19)	/	0.72 (0.36–1.38)	/

^a^ The values were represented as mean ± standard deviation or median (range). ^b^ the proportion vs. total controls. ^c^ published date. ^d^ the unit was nmol/g. ^e^ Grouped based on self-reported passive smoking. Abbreviations: No., number; gwk, gestational weeks; As, arsenic; Cd, cadmium; Cu, copper; Fe, iron; Hg, mercury; Mn, manganese; Pb, lead; Zn, zinc. ^f^ Data is missing.

## Data Availability

The data that support the findings of this study are available from the corresponding author upon reasonable request.

## References

[1] Chappell L.C., Cluver C.A., Kingdom J., Tong S. (2021). Pre-eclampsia. Lancet.

[2] Muglia L.J., Benhalima K., Tong S., Ozanne S. (2022). Maternal factors during pregnancy influencing maternal, fetal, and childhood outcomes. BMC Med..

[3] Miao J., Dou S., Shi T., Wang X., Wei X., Yan L., Ma B., Huang W., Zhang Y., Li S. (2023). Young adults’ blood selenium and lung function in Shandong province, China: A prospective cohort study. Innov. Med..

[4] Kahn L.G., Trasande L. (2018). Environmental toxicant exposure and hypertensive disorders of pregnancy: Recent findings. Curr. Hypertens. Rep..

[5] Poropat A.E., Laidlaw M.A.S., Lanphear B., Ball A., Mielke H.W. (2018). Blood lead and preeclampsia: A meta-analysis and review of implications. Environ. Res..

[6] Borghese M.M., Fisher M., Ashley-Martin J., Fraser W.D., Trottier H., Lanphear B., Johnson M., Helewa M., Foster W., Walker M. (2023). Individual, independent, and joint associations of toxic metals and manganese on hypertensive disorders of pregnancy: Results from the mirec Canadian pregnancy cohort. Environ. Health Perspect..

[7] Maduray K., Moodley J., Soobramoney C., Moodley R., Naicker T. (2017). Elemental analysis of serum and hair from pre-eclamptic south African women. J. Trace Elem. Med. Biol..

[8] Wang Y., Wang K., Han T., Zhang P., Chen X., Wu W., Feng Y., Yang H., Li M., Xie B. (2020). Exposure to multiple metals and prevalence for preeclampsia in Taiyuan, China. Environ. Int..

[9] El-Badry A., Rezk M., El-Sayed H. (2018). Mercury-induced oxidative stress may adversely affect pregnancy outcome among dental staff: A cohort study. Int. J. Occup. Environ. Med..

[10] Liu T., Zhang M., Guallar E., Wang G., Hong X., Wang X., Mueller N.T. (2019). Trace minerals, heavy metals, and preeclampsia: Findings from the Boston birth cohort. J. Am. Hear. Assoc..

[11] Rabinowitz M., Bellinger D., Leviton A., Needleman H., Schoenbaum S. (1987). Pregnancy hypertension, blood pressure during labor, and blood lead levels. Hypertension.

[12] Sandoval-Carrillo A., Méndez-Hernández E.M., Antuna-Salcido E.I., Salas-Pacheco S.M., Vázquez-Alaniz F., Téllez-Valencia A., Aguilar-Durán M., Barraza-Salas M., Castellanos-Juárez F.X., La Llave-León O. (2016). Arsenic exposure and risk of preeclampsia in a Mexican mestizo population. BMC Pregnancy Childbirth.

[13] Sowers M., Jannausch M., Scholl T., Li W., Kemp F.W., Bogden J.D. (2002). Blood lead concentrations and pregnancy outcomes. Arch. Environ. Health.

[14] Lewandowska M., Sajdak S., Marciniak W., Lubiński J. (2019). First trimester serum copper or zinc levels, and risk of pregnancy-induced hypertension. Nutrients.

[15] Liu T., Zhang M., Rahman M.L., Wang X., Hinkle S.N., Zhang C., Mueller N.T. (2021). Exposure to heavy metals and trace minerals in first trimester and maternal blood pressure change over gestation. Environ. Int..

[16] Ng S.W., Norwitz S.G., Norwitz E.R. (2019). The impact of iron overload and ferroptosis on reproductive disorders in humans: Implications for preeclampsia. Int. J. Mol. Sci..

[17] Buck Louis G.M., Yeung E., Sundaram R., Laughon S.K., Zhang C. (2013). The exposome--exciting opportunities for discoveries in reproductive and perinatal epidemiology. Paediatr. Perinat. Epidemiol..

[18] Schecter A., Lorber M., Guo Y., Wu Q., Yun S.H., Kannan K., Hommel M., Imran N., Hynan L.S., Cheng D. (2013). Phthalate concentrations and dietary exposure from food purchased in New York state. Environ. Health Perspect..

[19] Jin M., Chen H., Na J., An H., Li Z., Li N. (2022). Passive smoking and insomnia in rural Chinese nonsmoking housewives: An environmental and genetic perspective. Environ. Int..

[20] Ren M., Zhao J., Wang B., An H., Li Y., Jia X., Wang J., Wang S., Yan L., Liu X. (2022). Associations between hair levels of trace elements and the risk of preterm birth among pregnant women: A prospective nested case-control study in Beijing birth cohort (bbc), China. Environ. Int..

[21] Li Q.G., Liu G.H., Qi L., Wang H.C., Ye Z.F., Zhao Q.L. (2022). Heavy metal-contained wastewater in China: Discharge, management and treatment. Sci. Total Environ..

[22] Shahab A., Hui Z., Rad S., Xiao H., Siddique J., Huang L.L., Ullah H., Rashid A., Taha M.R., Zada N. (2023). A comprehensive review on pollution status and associated health risk assessment of human exposure to selected heavy metals in road dust across different cities of the world. Environ. Geochem. Health.

[23] Manduca P., Diab S.Y., Qouta S.R., Albarqouni N.M., Punamaki R.L. (2017). A cross sectional study of the relationship between the exposure of pregnant women to military attacks in 2014 in Gaza and the load of heavy metal contaminants in the hair of mothers and newborns. BMJ Open.

[24] Muniroh M., Bakri S., Gumay A.R., Dewantiningrum J., Mulyono M., Hardian H., Yamamoto M., Koriyama C. (2022). The first exposure assessment of mercury levels in hair among pregnant women and its effects on birth weight and length in semarang, central java, indonesia. Int. J. Environ. Res. Public Health.

[25] Trdin A., Snoj Tratnik J., Stajnko A., Marc J., Mazej D., Sešek Briški A., Kastelec D., Prpić I., Petrović O., Špirić Z. (2020). Trace elements and apoe polymorphisms in pregnant women and their new-Borns. Environ. Int..

[26] Palir N., Stajnko A., Snoj Tratnik J., Mazej D., Briški A.S., France-Štiglic A., Rosolen V., Mariuz M., Giordani E., Barbone F. (2023). Alad and apoe polymorphisms are associated with lead and mercury levels in Italian pregnant women and their newborns with adequate nutritional status of zinc and selenium. Environ. Res..

[27] Black A.P., Knight R., Batty J., Haswell S.J., Lindow S.W. (2002). An analysis of maternal and fetal hair lead levels. BJOG Int. J. Obstet. Gynaecol..

[28] Zhao R., Wu Y., Zhao F., Lv Y., Huang D., Wei J., Ruan C., Huang M., Deng J., Huang D. (2017). The risk of missed abortion associated with the levels of tobacco, heavy metals and phthalate in hair of pregnant woman: A case control study in Chinese women. Medicine.

[29] Zhu Y., Li Z., Pang Y., Huo W., Li N., Li Z., Zhang J., Ye R., Wang B. (2018). Association between chronic exposure to tobacco smoke and accumulation of toxic metals in hair among pregnant women. Biol. Trace Elem. Res..

[30] Kippler M., Gyllenhammar I., Glynn A., Levi M., Lignell S., Berglund M. (2021). Total mercury in hair as biomarker for methylmercury exposure among women in central sweden—A 23 year long temporal trend study. Environ. Pollut..

[31] Kocyłowski R., Lewicka I., Grzesiak M., Gaj Z., Sobańska A., Poznaniak J., von Kaisenberg C., Suliburska J. (2018). Assessment of dietary intake and mineral status in pregnant women. Arch. Gynecol. Obstet..

[32] Ripley S., Robinson E., Johnson-Down L., Andermann A., Ayotte P., Lucas M., Nieboer E. (2018). Blood and hair mercury concentrations among Cree first nations of Eeyou Istchee (Quebec, Canada): Time trends, prenatal exposure and links to local fish consumption. Int. J. Circumpolar Health.

[33] Sikorski R., Juszkiewicz T., Paszkowski T., Radomański T., Szkoda J., Milart P. (1986). Hair trace metal concentration of pregnant women at term in comparison with blood and milk levels. Eur. J. Obstet. Gynecol. Reprod. Biol..

[34] Dawson E.B., Evans D.R., Nosovitch J. (1999). Third-trimester amniotic fluid metal levels associated with preeclampsia. Arch. Environ. Health.

[35] Ikechukwu I.C., Ojareva O.I., Ibhagbemien A.J., Okhoaretor O.F., Oluwatomi O.B., Akhalufo O.S., Oluwagbenga A.T., Chigaekwu M.N. (2012). Blood lead, calcium, and phosphorus in women with preeclampsia in Edo state, Nigeria. Arch. Environ. Occup. Health.

[36] Vigeh M., Yokoyama K., Ramezanzadeh F., Dahaghin M., Sakai T., Morita Y., Kitamura F., Sato H., Kobayashi Y. (2006). Lead and other trace metals in preeclampsia: A case-control study in Tehran, Iran. Environ. Res..

[37] He J., Pu Y., Du Y., Liu H., Wang X., He S., Ai S., Dang Y. (2024). An exploratory study on the association of multiple metals in serum with preeclampsia. Front. Public Health.

[38] Huang C., Lai C., Xu P., Zeng G.M., Huang D.L., Zhang J.C., Zhang C., Cheng M., Wan J., Wang R.Z. (2017). Lead-induced oxidative stress and antioxidant response provide insight into the tolerance of *Phanerochaete chrysosporium* to lead exposure. Chemosphere.

[39] Laine J.E., Ray P., Bodnar W., Cable P.H., Boggess K., Offenbacher S., Fry R.C. (2015). Placental cadmium levels are associated with increased preeclampsia risk. PLoS ONE.

[40] Siddiqui I.A., Jaleel A., Kadri H.M., Saeed W.A., Tamimi W. (2011). Iron status parameters in preeclamptic women. Arch. Gynecol. Obstet..

[41] Gul A.Z., Atakul N., Selek S., Atamer Y., Sarıkaya U., Yıldız T., Demirel M. (2022). Maternal serum levels of zinc, copper, and thiols in preeclampsia patients: A case-control study. Biol. Trace Elem. Res..

[42] Rafeeinia A., Tabandeh A., Khajeniazi S., Marjani A.J. (2014). Serum copper, zinc and lipid peroxidation in pregnant women with preeclampsia in Gorgan. Open Biochem. J..

[43] Chen Y., Pu Y., Liu H., Cao A., Du Y., He S., Ai S., Dang Y. (2024). A study on the mediating role of serum hormones in the effects of heavy metals on preeclampsia. Environ. Pollut..

[44] Sarwar M.S., Ahmed S., Ullah M.S., Kabir H., Rahman G.K., Hasnat A., Islam M.S. (2013). Comparative study of serum zinc, copper, manganese, and iron in preeclamptic pregnant women. Biol. Trace Elem. Res..

[45] Lee P.K., Yu S., Jeong Y.J., Seo J., Choi S.G., Yoon B.Y. (2019). Source identification of arsenic contamination in agricultural soils surrounding a closed cu smelter, south Korea. Chemosphere.

[46] Mielcarek K., Puścion-Jakubik A., Gromkowska-Kępka K.J., Soroczyńska J., Karpińska E., Markiewicz-Żukowska R., Naliwajko S.K., Moskwa J., Nowakowski P., Borawska M.H. (2020). Comparison of zinc, copper and selenium content in raw, smoked and pickled freshwater fish. Molecules.

[47] Stratakis N., Conti D.V., Borras E., Sabido E., Roumeliotaki T., Papadopoulou E., Agier L., Basagana X., Bustamante M., Casas M. (2020). Association of fish consumption and mercury exposure during pregnancy with metabolic health and inflammatory biomarkers in children. JAMA Netw Open.

[48] White C.M. (2022). Lead in mineral or multivitamin-multimineral products. Ann. Pharmacother..

[49] Golding J., Steer C.D., Gregory S., Lowery T., Hibbeln J.R., Taylor C.M. (2016). Dental associations with blood mercury in pregnant women. Community Dent. Oral Epidemiol..

[50] Menezes-Filho J.A., Carvalho C.F., Rodrigues J.L.G., Araújo C.F.S., Dos Santos N.R., Lima C.S., Bandeira M.J., Marques B.L.S., Anjos A.L.S., Bah H.A.F. (2018). Environmental co-exposure to lead and manganese and intellectual deficit in school-aged children. Int. J. Environ. Res. Public Health.

[51] Obeng-Gyasi E. (2019). Sources of lead exposure in various countries. Rev. Environ. Health.

[52] Jiang M., Zhao H. (2024). Joint association of heavy metals and polycyclic aromatic hydrocarbons exposure with depression in adults. Environ. Res..

[53] Li Z., Kuang H., Li L., Wu M., Liao Z., Zeng K., Ye Y., Fan R. (2023). What adverse health effects will environmental heavy metal co-exposure bring us: Based on a biological monitoring study of sanitation workers. Environ. Sci. Pollut. Res. Int..

[54] Fu Z., Xu X., Cao L., Xiang Q., Gao Q., Duan H., Wang S., Zhou L., Yang X. (2024). Single and joint exposure of Pb, Cd, Hg, Se, Cu, and Zn were associated with cognitive function of older adults. Sci. Rep..

[55] Wells E.M., Herbstman J.B., Lin Y.H., Hibbeln J.R., Halden R.U., Witter F.R., Goldman L.R. (2017). Methyl mercury, but not inorganic mercury, associated with higher blood pressure during pregnancy. Environ. Res..

